# Institutional Notes and Queries

**Published:** 1902-10-18

**Authors:** 


### INSTITUTIONAL NOTES AND QUERIES.

*Training School for Teachers.*

Can you tell me if there is any other training school such as that mentioned in a sociology article which appeared in The Hospital in August?— *C. S.*

Until recently nothing in the way of a training school for teachers who had not passed through the normal colleges existed in London, but a training college has now been opened in the building of the London School of Economics, Clare Market, under the auspices of the London County Council. It is intended to give students a practical and thorough training such as will fit them to teach satisfactorily in elementary and secondary schools. There are sixty students, made up partly of King's scholars and partly of university graduates. The training college is attached to the University of London.

